# Association of VAMP5 and MCC genetic polymorphisms with increased risk of Hirschsprung disease susceptibility in Southern Chinese children

**DOI:** 10.18632/aging.101423

**Published:** 2018-04-25

**Authors:** Jinglu Zhao, Xiaoli Xie, Yuxiao Yao, Qiuming He, Ruizhong Zhang, Huimin Xia, Yan Zhang

**Affiliations:** 1 Department of Pediatric Surgery, Guangzhou Institute of Pediatrics, Guangzhou Women and Children’s Medical Center, Guangzhou Medical University, Guangzhou 510623, Guangdong, China; * Equal contribution

**Keywords:** Hirschsprung disease, association, *VAMP5*, *MCC*

## Abstract

Hirschsprung disease (HSCR) is a genetic disorder characterized by the absence of neural crest cells in parts of the intestine. This study aims to investigate the association of vesicle-associated membrane protein 5 (*VAMP5*) and mutated in colorectal cancer (*MCC*) genetic polymorphisms and their correlated risks with HSCR. We examined the association in four polymorphisms (rs10206961, rs1254900 and rs14242 in *VAMP5*, rs11241200 in *MCC*) and HSCR susceptibility in a Southern Chinese population composed of 1473 cases and 1469 controls. Two variants in *VAMP5* were replicated as associated with HSCR. Interestingly, we clarified SNPs rs10206961 and rs1254900 in *VAMP5* are more essential for patients with long-segment aganglionosis (LHSCR). Relatively high expression correlation was observed between *VAMP5* and *MCC* using data from public database showing there may exist potential genetic interactions. SNP interaction was cross-examined by logistic regression and multifactor dimensionality reduction analysis revealing that *VAMP5* rs1254900 and *MCC* rs11241200 were interacting significantly, thereby contributing to the risk of HSCR. The results suggest that significant associations of the rs10206961 and rs14242 in *VAMP5* with an increased risk of HSCR in Southern Chinese, especially in LHSCR patients. This study provided new evidence of epistatic association of *VAMP5* and *MCC* with increased risk of HSCR.

## Introduction

Hirschsprung disease (HSCR) is the most common cause of neonatal intestinal obstruction [1], defined by the partial or complete absence of the neural crest cells in the intestinal tract [2]. The overall prevalence of HSCR among the Asian population is estimated at 2.8/10,000 live births and displayed a significant racial variation [3].HSCR can be classified into three types based on the length of the aganglionic tract, including short-segment HSCR (S-HSCR), long-segment HSCR (L-HSCR) and total colonic aganglionosis (TCA) with the percentages around 80%, 15% and 5% respectively [4,5]. HSCR is a complex multifactorial disease, which is mainly determined by individual genetic factors [6,7]. The recurrence risk in siblings varied from 1% to 33% depending on the length of the aganglionic segments and gender of the probands. More than ten genes were identified as contributed to the pathogenesis of HSCR including *RET, GDNF, EDNRB, EDN3* and so on [8]. However, mutations in these genes account for only ∼50% of the known cases of HSCR [9].

Vesicle-associated membrane protein 5 (*VAMP5*) has been reported to provide their subcellular targeting in the synaptic vesicle fusion process of enteric nervous system (ENS) neurotransmission [10,11]. Shin JG et al. suggested that two single nucleotide polymorphisms (SNP, rs10206961 and rs1254900) of *VAMP5* were the potential risk locus in TCA progression by using 187 Korean HSCR patients and 283 unaffected controls (P=0.006 for rs10206961, P=8.03×10^-5^ for rs1254900), we also included SNP rs14242 in *VAMP5* with suggestive significance to HSCR (P=0.04) for further replication [12].

Mutated in colorectal cancer (*MCC*) gene which is located on chromosome 5q21 encodes a protein comprised of 829 amino acids [13]. *MCC* is a tumor suppressor in different types of cancers, including hepatocellular carcinoma, colorectal cancer (CRC) and acute myeloid leukemia [14]. MCC is strongly related to the plasma membrane and membrane organelles in fmouse intestinal epithelial cells and neuronal cells via an immunoelectron microscopic analysis [15]. Additionally, case-control studies suggested that *MCC* might confer alterative genetic susceptibility to CRC in individuals with schizophrenia, implying *MCC* as a factor related to neurodevelopmental disorders [16]. A genome-wide association study (GWAS) by Garcia-Barcelo et al*.* has revealed that *MCC* was a plausible candidate gene of HSCR [17]. Rs11241200 in *MCC* was chosen for replication in current study according to this GWAS. It is noteworthy that *MCC* did not only function as an independent tumor suppressor in the majority of colorectal cancers, but also functioned as a susceptibility gene in HSCR.

The underlying genes remain largely unknown, especially the interplays between these susceptibility genes. The aim of this study was to ascertain whether the genetic polymorphisms of *VAMP5* (rs10206961, rs1254900 and rs14242) and *MCC* (rs11241200) were associated with HSCR in 1470 Chinese HSCR cases and 1473 controls. We have clarified SNPs rs10206961 and rs1254900 in *VAMP5* which are more essential for patients with LHSCR. Interestingly, further elaborated of SNP rs1254900 (*VAMP5*) and rs11241200 (*MCC*) were found to be interacted significantly, thereby contributing to the risk of SHSCR. This finding may add *MCC* to the list of genes as associated with HSCR. Further replications and functional evaluations are still required.

## RESULTS

### Association of *VAMP5* and *MCC* SNPs with HSCR

Four SNPs were selected for replication in this study including 3 SNPs on *VAMP 5* and 1 SNPs on *MCC*. The selection criteria were detailed listed in the Method. Detailed information about four SNPs genotyped in this study was shown in Table 1 using 1473 HSCR patients and 1469 HSCR-free controls from South China. The genotype distribution for the 4 SNPs followed the hardy-weinberg equilibrium (HWE) in the control subjects *(P_hwe*=0.58 for rs10206961, *P_hwe*=0.46 for rs1254900, *P_hwe*=0.07 for rs14242 and P_*hwe*=0.07 for rs11241200). All four SNPs are located in the intronic region. SNPs rs10206961 and rs14242 in *VAMP5* shows significant association with HSCR (0.044≤P_adj≤0.046, 1.12≤OR≤1.17 for rs10206961; P_adj=0.020, OR=1.20 for rs14242). Inconsistent with the report by Shin JG et al. [12] for the SNP rs1254900 in *VAMP5*, we failed to replicate the association in our population (0.666≤P≤0.953, 0.97≤OR≤1.04 for rs1254900). For the most prominent SNP rs11241200 in *MCC*, 0.282≤P≤0.438 for rs11241200, we failed to replicate the association as presented previously [17]. To better digest the effective pattern for the two SNPs on *VAMP5*, we specified the samples following four different genetic models including additive, dominant, recessive and genotypic models. Larger effect was observed in both SNPs following dominant models (OR=1.12/1.17 for 10206961; OR=1.20 for 14242 respectively).

**Table 1:** Replication results of *VAMP5* and *MCC* SNPs in Southern Chinese children.

CHR	SNP	gene	feature	left_gene	right_gene	BP	A1/A2	Case	Control	P		OR
								1469	1473	*P_raw*	*P_adj*	
*P_hwe=0.58*												
2	rs10206961	*VAMP5*	intron[NM_006634.2]	*VAMP8*	*RNF181*	85587861	T/C	1159/1699	1108/1802	0.054		1.11(1.00~1.23)
							FREQ	0.41	0.38	-	-	-
							ADD	235/689/505	216/676/563	0.056	**0.044**	1.12(1.00~1.24)
							DOM	924/505	892/563	0.062	**0.046**	1.17(1.00~1.37)
							REC	235/1194	216/1239	0.237	0.232	1.13(0.92~1.39)
*P_hwe=0.46*												
2	rs1254900	*VAMP5*	intron[NM_006634.2]	*VAMP8*	*RNF181*	85589211	A/G	1371/1475	1395/1513	0.878		1.01(0.91~1.12)
							FREQ	0.48	0.48	-	-	-
							ADD	327/717/379	342/711/401	0.879	0.953	1.00(0.90~1.12)
							DOM	1044/379	1053/401	0.569	0.666	1.04(0.88~1.23)
							REC	327/1096	342/1112	0.731	0.722	0.97(0.81~1.16)
*P_hwe=0.07*												
2	rs14242	*VAMP5*	intron[NM_006634.2]	*VAMP8*	*RNF181*	85593289	T/C	874/1968	840/2070	0.118		1.09(0.98~1.23)
							FREQ	0.31	0.29	-	-	-
							ADD	127/620/674	135/570/750	0.12	0.081	1.11(0.99~1.24)
							DOM	747/674	705/750	0.027	**0.02**	1.20(1.03~1.39)
							REC	127/1294	135/1320	0.751	0.912	0.99(0.76~1.28)
*P_hwe=0.07*												
5	rs11241200	*MCC*	intron[NM_001085377.1]	*DCP2*	*TSSK1B*	113339402	G/T	376/1846	346/1816	0.413		1.07(0.91~1.25)
							FREQ	0.17	0.16	-	-	-
							ADD	42/292/777	36/274/771	0.427	0.282	1.09(0.93~1.28)
							DOM	334/777	310/771	0.476	0.336	1.10(0.91~1.32)
							REC	42/1069	36/1045	0.57	0.438	1.20(0.76~1.91)

CHR: Chromosome; SNP: Single Nucleotide Polymorphism; BP: Base pair of where the SNP is located. Func.refgene: The function role of SNP in the gene. Gene.refgene: The gene where the SNP located to; A1/A2 indicates the risk allele and protective allele to disease; Freq: indicates risk allele frequency of the SNP in cases or controls. ADD, DOM and REC indicate the association test following additive, dominant, recessive. The P value indicates the significance based on different genetic models. *P_raw* was the original p-values. *P_adj* was calculated adjusting the genders of all the samples. The calculation of odds ratio (OR) is also based on the risk allele of each SNP.

### Independence test of the replicated SNPs

**Figure 1:**
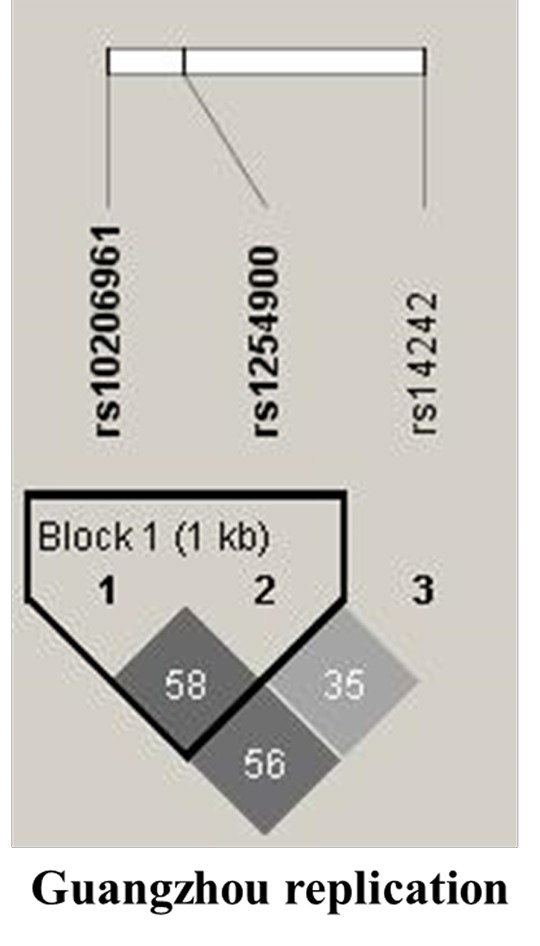
**The linkage disequilibrium patterns (LD) of the SNPs in *VAMP5* included in this study.** Haplotype blocks in *VAMP5* were defined according to the LD value in Guangzhou replication. The numbers in the boxes are the pairwise correlation coefficient r^2^ between respective SNPs. Darker shades of gray indicate higher value of LD. Lighter shades of gray represents lower value of LD. The highest LD value of 0.58 was observed between rs10206961 and rs1254900, while rs14242 showed limited LD with all the other SNPs among these populations.

To further identify the independence of SNPs previous replicated in *VAMP5*, the linkage disequilibrium (LD) patterns were examined on Guangzhou replication data (Figure 1). Consistent with the public data as shown in Supplementary Figure 1, SNPs rs10206961 and rs1254900 showed moderate LD with each other (r^2^ =0.58). SNP rs14242 showed limited LD with all the other SNPs (r^2^ =0.35).

Conditional logistic regression analysis was performed to investigate the independent effects among the three SNPs in VAMP5 (Table 2). SNPs rs10206961and rs14242 kept significant after controlling the effect of rs1254900 (P=2.3E-03, OR=1.30 for rs10206961; P=0.022, OR=1.18 for rs14242). SNPs rs10206961 remains no significance to disease if the effect of rs14242 were controlled, vice versa (P=0.48, OR=1.06 for rs10206961 P=0.45, OR=1.07 for rs14242). Interestingly, though SNP rs1254900 showed no evidence of association to disease (Table 1), we observed SNP rs1254900 was significant if the effect of SNP rs10206961 was adjusted (P=0.018, OR=1.22), These results raised up the notion that diversified effects of these SNPs exist in this region for disease susceptibility, such as epistatic effect.

**Table 2:** Independence testing among SNPs in *VAMP5*.

NP	SNP whose effect were adjusted		
	rs10206961	rs1254900	rs14242
rs10206961	NA	**P=2.3E-03**	P=0.48
		**1.30(1.01~1.55)**	1.06(0.90~1.25)
rs1254900	**P=0.018**	NA	P=0.17
	**1.22(1.04~1.45)**		1.10(0.96~1.25)
rs14242	P=0.45	**P=0.022**	NA
	1.07 (0.90~1.27)	**1.18(1.03~1.36)**	

SNP: Single Nucleotide Polymorphism; The data in each column represent the remaining effect of association (P-values) after adjusting for the effect of SNP(s) on the top row of each Column.

### Intergenic SNPs show epistatic effect to HSCR

As shown in Table 3, pairwise epistasis test was among the three SNPs in *VAMP5*. Surprisingly, we failed to observe any intragenic epistatic association to disease (0.11≤P≤0.5, 0.86≤OR≤1.15). As presented above, *VAMP5* and *MCC* may involve in the enteric nervous system (ENS) neurotransmission. We also observed the two genes showed relative high coexpression correlation in Peripheral blood mononuclear cell (PBMC) with confidence (P=0.002, OR=1.26) using the data extracted from ImmuCo [18]. Thus, we also included the SNP in MCC (rs11241200), the result suggested significant elevated effect between SNP rs1254900 (*VAMP5*) and rs11241200 (*MCC*) to disease (P_int= 2.9E-03, OR=1.42). SNP rs1254900, rs14242 showed less synergetic effect with other SNPs reflected by the insignificant P values shaded in grey. Another statistical method for testing epistatic interaction was also applied for further validation. Pairwise Multifactor dimensionality reduction (MDR) analysis was adopted here to test the epistatic interaction between SNP pairs. Table 3 showed the results of cross-validation consistency (CVC) and Balanced accuracy (BA) obtained from MDR analysis of the two-locus model, which showed significant pairwise interactions. The significance of the result was tested showing the consistent higher effect size between epistatic rs11241200 (*MCC*) with rs10206961, rs1254900 and rs14242 (*VAMP5*) to disease (*P*=0.002, OR=1.26).

**Table 3:** Pair-wise epistatic interacting results among *VAMP5* and *MCC* SNPs done by logistic regression and Multifactor dimensionality reduction (MDR).

SNP			*VAMP5*			*MCC*
			rs10206961	rs1254900	rs14242	rs11241200
			logistic regression			
*VAMP5*	rs10206961	MDR	NA	P_int= 0.50	P_int= 0.11	P_int= 0.15
				1.06 (0.89~1.26)	0.86 (0.72~1.03)	0.84 (0.67~1.06)
	rs1254900			NA	P_int= 0.15	**P_int= 2.9E-03**
					1.15 (0.95~1.39)	**1.42 (1.14~1.79)**
	rs14242				NA	P_int= 0.07
						0.81 (0.64~1.02)
*MCC*	rs11241200			CVC^a^=10/10 BA^b^=0.53		NA
				P=0.002 1.26(1.09~1.46)		

SNP: Single Nucleotide Polymorphism; a Cross-validation consistency (CVC) reflects the number of times MDR analysis identified the same model as the data were divided into different segments. b Balanced accuracy (BA) is defined as (sensitivity + specificity)/2.

### Clinical stratification of SNPs in *VAMP5* and *MCC* with HSCR

HSCR is heterogenous varied with different subclinical manifestations, typically classified by the aganglionisis length of the patients including SHSCR, LHSCR and TCA patients. For the two SNP including rs14242 and rs10206961 which showed evidence of association to HSCR in current study, rs14242 is associated with increased risk of the SHSCR though the effect is marginal (P=0.039, OR=1.19). SNP rs10266961 showed much stronger evidence of association to LHSCR with larger effect size and more stringent P value (2.97E-03≤P≤0.027, 0.61≤OR≤1.44), especially following the additive model. Surprisingly, despite from the epistatic effect with SNP rs1254900 to disease, we also observed a significant individual association to LHSCR patients. (P=4.62E-03, OR=0.61), highlighting extra roles for the SNP to disease. So we further examined the epistatic effect of SNP rs11241200 (*MCC*) and rs1254900 (*VAMP5*) to different HSCR patients. As shown in Table 4, we only observed significant epistatic associations in SHSCR patients to the disease (P_int=8.9E-03, OR=1.40), for LHSCR patients, marginal effect was observed which may be caused by limited samples waiting for further replications (P_int=0.066, OR=1.48). It is also possible SNP rs11254900 plays multiple roles leading to disease association which also required functional characterization.

**Table 4:** HSCR subphenotype interaction examination among rs1254900 (*VAMP5*) and rs11241200 (*MCC*).

SNP		*MCC*		
		SHSCR	LHSCR	TCA
		rs11241200		
		logistic regression		
*VAMP5*	rs1254900	P_int=8.9E-03	P_int= 0.066	P_int= 0.772
		1.40(1.09~1.81)	1.48(0.97~2.06)	1.09(0.62~1.92)

HSCR: Hirschsprung disease; SNP: Single Nucleotide Polymorphism; SHSCR: aganglionosis length including short-length LHSCR: long-length; TCA: total colonic aganglionosis.

## DISCUSSION

HSCR is a complex clinical syndrome. Increasing studies focused on case-control and trio based study designs. It the largest population-based study to the correlation of *VAMP5* and *MCC* genetic polymorphisms with HSCR risk in our study that 1473 cases and 1470 unrelated controls were enrolled in. We have replicated two SNPs rs10206961 and rs14242 in *VAMP5* that were associated with HSCR (Tables 1, 2). In further subclinical manifestation analysis, we further clarified SNPs rs10206961 and rs1254900 in *VAMP5* and found they were essential for patients with LHSCR (Tables 3, 5). Interestingly, SNP interaction confirmed through logistic regression and multifactorial dimensionality reduction analysis revealed that the genotypes of the polymorphisms of *VAMP5* rs1254900 and *MCC* rs11241200 were interacting significantly, thereby contributing to the risk of SHSCR (Table 4).

**Table 5:** The association results of *VAMP5* and *MCC* SNPs to different subclinical features classified by aganglionosis length including short-length (SHSCR), long-length (LHSCR) and total colonic aganglionosis (TCA) .

	CHR	SNP	BP	A1/A2	TEST	Subphenotype	Control	P	OR
SHSCR	2	rs10206961	85587861	T/C	ADD	152/486/364	216/676/563	0.357	1.06(0.94~1.19)
					DOM	638/364	892/563	0.217	1.11(0.94~1.32)
					REC	152/850	216/1239	0.919	1.01(0.80~1.28)
	2	rs1254900	85589211	A/G	ADD	249/497/256	342/711/401	0.249	1.07(0.95~1.20)
					DOM	746/256	1053/401	0.300	1.10(0.92~1.33)
					REC	249/753	342/1112	0.398	1.09(0.90~1.32)
	2	rs14242	85593289	T/C	ADD	83/444/474	135/570/750	0.217	1.08(0.95~1.23)
					DOM	527/474	705/750	**0.039**	1.19(1.01~1.41)
					REC	83/918	135/1320	0.413	0.88(0.66~1.19)
	5	rs11241200	1.13E+08	G/T	ADD	32/206/539	36/274/771	0.198	1.12(0.94~1.34)
					DOM	238/539	310/771	0.270	1.12(0.91~1.38)
					REC	32/745	36/1045	0.296	1.31(0.79~2.16)
LHSCR	2	rs10206961	85587861	T/C	ADD	58/142/89	216/676/563	**2.97E-03**	1.32(1.10~1.57)
					DOM	200/89	892/563	**8.98E-03**	1.44(1.10~1.89)
					REC	58/231	216/1239	**0.027**	1.44(1.04~1.99)
	2	rs1254900	85589211	A/G	ADD	45/154/85	342/711/401	**0.024**	0.81(0.67~0.97)
					DOM	199/85	1053/401	0.365	0.88(0.66~1.16)
					REC	45/239	342/1112	**4.62E-03**	0.61(0.43~0.86)
	2	rs14242	85593289	T/C	ADD	27/119/135	135/570/750	0.325	1.10(0.91~1.34)
					DOM	146/135	705/750	0.247	1.16(0.90~1.51)
					REC	27/254	135/1320	0.819	1.05(0.68~1.63)
	5	rs11241200	1.13E+08	G/T	ADD	5/54/169	36/274/771	0.370	0.88(0.66~1.17)
					DOM	59/169	310/771	0.440	0.88(0.63~1.22)
					REC	5/223	36/1045	0.477	0.71(0.27~1.83)
TCA	2	rs10206961	85587861	T/C	ADD	14/33/35	216/676/563	0.903	0.98(0.71~1.35)
					DOM	47/35	892/563	0.532	0.87(0.55~1.36)
					REC	14/68	216/1239	0.534	1.21(0.67~2.19)
	2	rs1254900	85589211	A/G	ADD	23/35/23	342/711/401	0.683	1.07(0.78~1.46)
					DOM	58/23	1053/401	0.783	0.93(0.57~1.53)
					REC	23/58	342/1112	0.334	1.28(0.78~2.11)
	2	rs14242	85593289	T/C	ADD	10/32/41	135/570/750	0.450	1.14(0.82~1.58)
					DOM	42/41	705/750	0.645	1.11(0.71~1.73)
					REC	10/73	135/1320	0.362	1.38(0.69~2.73)
	5	rs11241200	1.13E+08	G/T	ADD	3/23/40	36/274/771	0.066	1.48(0.97~2.25)
					DOM	26/40	310/771	0.056	1.65(0.99~2.76)
					REC	3/63	36/1045	0.537	1.46(0.44~4.91)

CHR: Chromosome; SNP: Single Nucleotide Polymorphism; BP: Base pair of where the SNP is located. Func.refgene: The function role of SNP in the gene. Gene.refgene: The gene where the SNP located to; A1/A2 indicates the risk allele and protective allele to disease; Freq: indicates risk allele frequency of the SNP in cases or controls. ADD, DOM and REC indicate the association test following additive, dominant, recessive and genotypic models. The P value indicates the significance based on different genetic models. The calculation of odds ratio (OR) is also based on the risk allele of each SNP.

*VAMP5* belongs to part of the vesicle-associated membrane protein (VAMP) and the soluble NSF attachment protein receptor (SNARE) superfamily. SNARE superfamily is responsible for the last stage of docking and subsequent fusion in diverse intracellular membrane transport events [19]. *VAMP5* can provide their subcellular targeting in neurotransmission [11,19]. It may also facilitate glucose transporter type 4 (GLUT-4) translocation from the intracellular pool to the plasma membrane. Downregulation of *VAMP5* might determine reduced GLUT4 membranal expression, followed by reduced glucose transport [20]. Accordingly, we hypothesize that the *VAMP5* risk alleles could unbalance the metabolism of its encoded protein leading to the disorders of intestinal protein synthesis, although there is a lack of direct experimental support for this assumption. Considering the facts that pathophysiology of HSCR, it is caused by a congenital absence of neurons in a portion of the intestinal tract. More specifically, genetic variants of *VAMP5* may functionally hinder the normal the migration and proliferation of enteric neural crest cell. Similar genetic studies have suggested that there was a relationship between *VAMP5* polymorphisms and HSCR [12]. Three SNPs in *VAMP5* overlaps with the previously reported potential association of *VAMP5* polymorphisms with 21 TCA patients [20]. But in our study, we observed the association of VAMP5 with HSCR, especially in SHSCR and LHSCR patients, but we failed to replicate the association of SNPs using 82 TCA patients. The possible sources of this discrepancy could be attributed to number of samples. As shown in independence test result, SNP rs14242 showed limited LD with all the other SNPs (Figure 1, r^2^=0.35). Besides, our study presents the epistatic association between *MCC* and Vamp5. *MCC* is known to reduced activation of NF-κB signalling in colorectal cells as well as a factor related to neurodevelopmental disorders [21]. A study on mice has shown that *MCC* binds SH3/ankyrin domain gene 3 isoform were able to participate in neurodevelopmental, neurobehavioral and autism spectrum disorders [22]. Epistasis between the different genes provides us a perspective for disease etiology, not only limited HSCR, we also observed in other complex diseases such as colorectal cancer [23]. Taking the advantage of large replication samples, we tested the pairwise genetic epistasis between *VAMP5* and *MCC*. Intriguingly, a significant synergetic interaction between rs1254900 (*VAMP5*) and rs11241200 (*MCC*) was identified through the cross-validation by logistic regression and MDR analysis, thereby contributing to the risk of SHSCR. We could speculate that *VAMP5* and *MCC* gene are associated with the neurotransmitter release process that affects neurogenesis SNPs for the subphenotype interaction examination to make a more precise and convictive assessment, pointing to the increased risk of SHSCR comparing to other related diseases.

There are several limitations to this study. First, the effect of gene-environment interactions was not emphasized. Second, more accurate ORs should be adjusted by patient factors such as medication consumption and other exposure factors. Third, as the heterogeneity in different ethnicities influenced the results significantly, the findings from the Asian based studies were not convictive enough. We also calculated the power of current study using Epistasis Power Calculator (https://gump.qimr.edu.au/general/manuelF/epistasis/epipower4i.html), based on current sample size with the incidence rate 1 per 5000 infants, the power to detect pairwise epistatic effect is limited (0.61 for case-only study, 0.37 for case-control study), further replication in independent cohort was still required. Fourth, although this is the largest population-based study conducted to-date, the statistical power was still limited due to the relative insufficient sample size. Replication studies from other hospital with a larger sample size are encouraged to confirm the association. Lastly, the functional mechanisms subject to the association of *VAMP5* and *MCC* for HSCR is required in the further study, borrowing the idea from other diseases [24,25].

In summary, our study indicated significant associations between rs1254900 (*VAMP5*) and rs11241200 (*MCC*) as independently related with SHSCR status. We proposed a relationship that may fill the gap between genetic susceptibility and subclinical manifestation. These conclusions also will provide a basis for future efforts to understand the detailed mechanisms of this intestinal disorder. SNPs associated with increased severity or worsening progression of HSCR would potentially afford a better individualized treatment of patients.

## MATERIALS AND METHODS

### Study subjects

Following ethical approval by the institutional review board of Guangzhou Women and Children’s medical center, 1470 Southern Chinese HCSR cases (from 2000-2015; age range 8.37 ± 20.50 months; 83.67% males) were diagnosed with HSCR by surgical procedures and followed up histological examination. This pathologic evaluation of a rectal biopsy that shows nerve fibers in either mucosa or submucosa and the absence of ganglion cells in the submucosa [26]. All the cases were divided into three subgroups according to the segment lengths of aganglionosis including 1033 S-HSCR, 294 L-HSCR and 82 TCA. 1473 population control samples (age range 18.61 ± 19.75 months; 34.35% males) were collected with no history of HSCR and neurological disorders. Parental informed consent was obtained from all patient subjects in this study. The detailed clinical information of this study was summarized in Supplementary Table 1.

### SNP Genotyping and quality control

Three SNPs in *VAMP5* involved in the study were selected according to a GWAS in 187 Korean HSCR cases (Supplementary Table 2). Five SNPs were replicated in their study as shown in Supplementary Figure 1, two SNPs were removed according to the high LD (r^2^>0.9,rs1561198 and rs55971080) in Asians (the LD in CEU among the SNPs was less tightly with r^2^>0.8). The three SNPs with high LD were annotated by the RegulomeDB database (http://www.regulomedb.org/) to estimate the potential functional roles. The potential regulatory SNP (rs10206961) was remained for further replication.

Three SNPs in MCC from the HSCR GWAS by Garcia-Barcelo et al. (Supplementary Table 3) [17]. showed similar likelihood and effect size of disease association, linkage disequilibrium (LD) was examined among them reflecting the association might derive from one signal (r^2^>0.9). SNP rs11241200 was chosen for replication in current study with higher annotation score by Regulome DB [17]. Four selected SNPs were genotyped by MassARRAY iPLEX Gold system (Sequenom) on the samples. Hardy-Weinberg equilibrium tests were performed and SNPs with *P <* 0.05 were excluded from the final analysis. Quality control of the four SNPs was performed as follows: 1. Cases/controls were excluded from the analysis on the basis of SNPs with >10% missing data. 2. Any subjects with 10% missing call were removed. After all quality control procedures, all four SNPs were kept for further analysis consisted of 1470 cases and 1473 controls.

### Association analysis and subphenotype analysis

The SNPs were tested for associations with the disease by means of comparison of the minor allele frequency in cases and controls (basic allelic test) as well as other tests using PLINK1.9 (logistic regression for additive model, test of dominant and recessive models) [27]. Subphenotype association analyzes were performed by comparing cases with and without a certainly given subphenotype.

### Independence testing

Linkage disequilibrium (LD) patterns were analyzed and displayed by HaploView [28]. Logistic regression tests were performed using SNPTEST v2.5b [29]. Tests of independent contributions toward disease associations for SNPs in a single locus were done using logistic regression, adjusting for the effect of a specific SNP in the same locus.

### Gene coexpression

We visited ImmuCo, a database of gene Co-expression and Correlation in multiple cells including expression data for a total of 20,283 human (http://immuco.bjmu.edu.cn/) and examined the pairwise expression correlation between VAMP5 and MCC. The correlation was calculated by Graphpad 5.0

### Genetic epistasis

Epistasis test (case-control analysis) by logistic regression was adopted here for the parametric analysis of genetic interaction using PLINK1.9 [30]. PLINK uses a model according to allele dosage ranging from 0 to 2 indicating the number of risk alleles for each SNP, A and B, and fits the model in the form of Y =b_0_ + b_1_ SNPA + b_2_ SNPB +b_3_ SNPA*SNPB + e. The parameters b1, b2 and b3 indicate the contribution of SNP A and SNP B and interaction between A and B. The test for interaction is based on the coefficient b3. P values less than 0.05 were considered statistically significant.

Multifactor dimensionality reduction (MDR) was used to determine the genetic model that could most successfully predict the disease status or phenotype from several loci. Pairwise non-parametric epistasis test was also applied using MDR analysis [31]. This method includes a combined cross-validation (CV)/permutation testing procedure that minimizes false positive results by multiple examinations of the data. We determined the statistical significance by comparing the average prediction error from the observed data with the distribution of average prediction errors under the null hypothesis. The MDR analysis was carried out using version 2.0 of the open-source MDR software package that is freely available online (http://www.epistasis.org) [32].

## Supplementary Material

Supplementary File
