# Supplementary material for: Association of VAMP5 and MCC genetic polymorphisms with increased risk of Hirschsprung disease susceptibility in Southern Chinese children
Source: Aging (Albany NY). 2018 Apr 25;10(4):689–700. doi: 10.18632/aging.101423 (PMC5940112; doi:10.18632/aging.101423)
Supplement: Supplementary File [file aging-10-101423-s001.pdf]

SUPPLEMENTARY MATERIAL

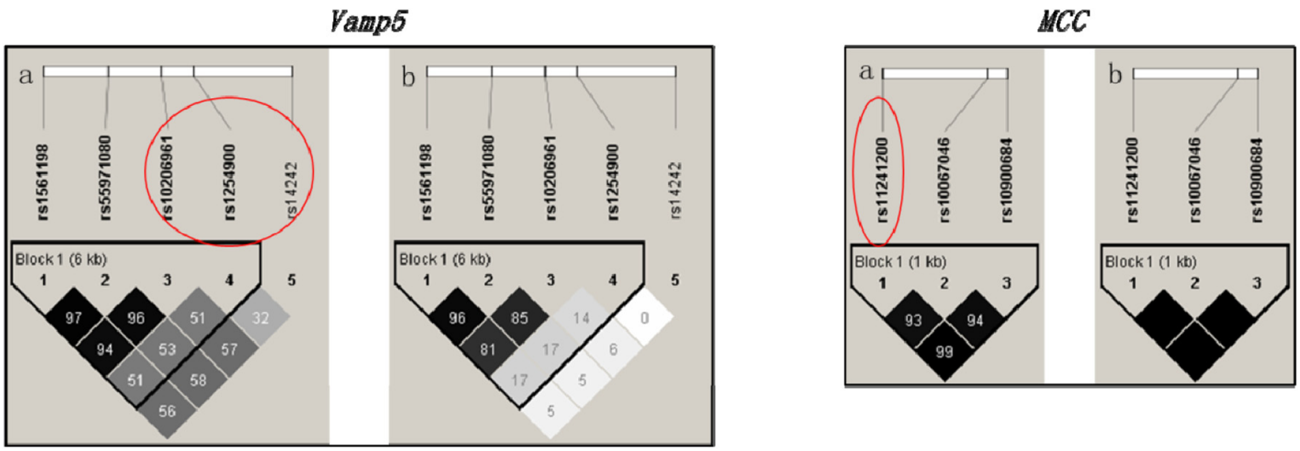

Supplementary Figure 1. The linkage disequilibrium patterns of the susceptibility single-nucleotide polymorphisms in *VAMP5* and *MCC* for Hirschsprung disease (HSCR) in different populations. CHB: Han Chinese. JPT: Japanese. CEU: Utah residents with ancestry from northern and western Europe. The LD (r2) patterns of five SNPs in *VAMP5* in CHB & JPT populations (a), CEU populations (b) from 1000G data. The LD (r^2) patterns of three SNPs in *MCC* in CHB & JPT populations (a), CEU populations (b) from 1000G data. The numbers in the boxes are the pairwise correlation coefficient r^2 between respective SNPs. Darker shades of color indicate higher value of LD. Lighter shades of color represents lower value of LD. In the previous HSCR genome-wide association study (GWAS), the red circle in left figure indicates three SNPs (rs10206961, rs1254900 and rs14242) in *VAMP5* with the most significant most significant P-values association with risk of HSCR. The red circle in right figure (rs11241200) was included for analysis because it has been reported to be associated with HSCR in the HSCR GWAS by Garcia-Barcelo et al.

Supplementary Table 1. The subclinical information collected for the subjects in this study.

HSCR subphenotype	Cases (n=1470)	%	Controls (n=1473)	%
Subjects				
Age range (Months)	8.37±20.50		18.61±19.75	
≤2	725	49.32%	458	31.09%
>2	745	50.68%	1015	68.91%
Gender				
Females	240	16.33%	967	65.65%
Males	1230	83.67%	506	34.35%
Clinical manifestation				
SHCSR	1033	70.27%		
LHCSR	294	20.00%		
TCA	82	5.58%		
Total intestine	3	0.20%		

HSCR: Hirschsprung disease; SHSCR: aganglionosis length including short-length; LHSCR: long-length; TCA: total colonic aganglionosis.

Supplementary Table 2. Shin et al, 2016 /283 Controls and 374 Cases

SNP	A1/A2	Control	HSCR	OR	P*
rs10206961	T/C	0.323	0.409	1.48（1.12~1.95）	0.007
rs1254900	T/C	0.495	0.455	0.83（0.63~1.10）	0.2
rs14242	A/G	0.293	0.358	1.36（1.02-1.80）	0.03

SNP: Single Nucleotide Polymorphism; A1/A2 indicates the risk allele and protective allele to disease; HSCR: Hirschsprung disease; The calculation of odds ratio (OR) is also based on the risk allele of each SNP. The P value indicates the significance based on different genetic models.

Supplementary Table 3. Garcia-Barcelo et al, 2009/346 Controls and 181 Cases.

SNP	A1/A2	Control	HSCR	OR	P
rs10900684	T/C	—	—	—	2.42E-05
rs11241200	A/C	—	—	—	3.45E-05
rs10067046	C/G	—	—	—	3.00E-04

SNP: Single Nucleotide Polymorphism; A1/A2 indicates the risk allele and protective allele to disease; HSCR: Hirschsprung disease; The calculation of odds ratio (OR) is also based on the risk allele of each SNP. The P value indicates the significance based on different genetic models.
